# Understanding Plant-Microbe Interactions for Phytoremediation of Petroleum-Polluted Soil

**DOI:** 10.1371/journal.pone.0017961

**Published:** 2011-03-18

**Authors:** Ming Nie, Yijing Wang, Jiayi Yu, Ming Xiao, Lifen Jiang, Ji Yang, Changming Fang, Jiakuan Chen, Bo Li

**Affiliations:** 1 Coastal Ecosystems Research Station of the Yangtze River Estuary, Ministry of Education Key Laboratory for Biodiversity Science and Ecological Engineering, Institute of Biodiversity Science, Fudan University, Shanghai, China; 2 The Institute of Global Environmental Change Research, Fudan University, Shanghai, China; 3 Center for Watershed Ecology, Institute of Life Science and Key Laboratory of Poyang Lake Environment and Resource Utilization, Nanchang University, Nanchang, Jiangxi, China; 4 College of Life and Environment Sciences, Shanghai Normal University, Shanghai, China; Cinvestav, Mexico

## Abstract

Plant-microbe interactions are considered to be important processes determining the efficiency of phytoremediation of petroleum pollution, however relatively little is known about how these interactions are influenced by petroleum pollution. In this experimental study using a microcosm approach, we examined how plant ecophysiological traits, soil nutrients and microbial activities were influenced by petroleum pollution in *Phragmites australis,* a phytoremediating species. Generally, petroleum pollution reduced plant performance, especially at early stages of plant growth. Petroleum had negative effects on the net accumulation of inorganic nitrogen from its organic forms (net nitrogen mineralization (NNM)) most likely by decreasing the inorganic nitrogen available to the plants in petroleum-polluted soils. However, abundant dissolved organic nitrogen (DON) was found in petroleum-polluted soil. In order to overcome initial deficiency of inorganic nitrogen, plants by dint of high colonization of arbuscular mycorrhizal fungi might absorb some DON for their growth in petroleum-polluted soils. In addition, through using a real-time polymerase chain reaction method, we quantified hydrocarbon-degrading bacterial traits based on their catabolic genes (i.e. *alkB* (alkane monooxygenase), *nah* (naphthalene dioxygenase) and *tol* (xylene monooxygenase) genes). This enumeration of target genes suggests that different hydrocarbon-degrading bacteria experienced different dynamic changes during phytoremediation and a greater abundance of *alkB* was detected during vegetative growth stages. Because phytoremediation of different components of petroleum is performed by different hydrocarbon-degrading bacteria, plants’ ability of phytoremediating different components might therefore vary during the plant life cycle. Phytoremediation might be most effective during the vegetative growth stages as greater abundances of hydrocarbon-degrading bacteria containing *alkB* and *tol* genes were observed at these stages. The information provided by this study enhances our understanding of the effects of petroleum pollution on plant-microbe interactions and the roles of these interactions in the phytoremediation of petroleum-polluted soil.

## Introduction

Phytoremediation is the use of plants and their associated microbes for environmental cleanup [1]. This technology is increasingly becoming an important approach in environmental and ecological research owing to its cost-effective and environmentally-friendly features [2]–[4]. The efficiency of phytoremediation depends mostly on the establishment of robust plant-microbe interactions [5]. Plants, through their ‘rhizosphere effects’, support hydrocarbon-degrading microbes that assist in phytoremediation in the root zone [6], [7]. For example, root activities in perennial ryegrass and alfalfa increase the number of rhizobacteria capable of petroleum degradation in the soil [2]. In turn, healthy microbial communities enhance soil nutrient availability to the plants [5], [8]. However, petroleum hydrocarbons are known to be harmful not only to plant growth and development, but also to microbial processes [9]–[12]. This is because petroleum hydrocarbons negatively affect photosynthesis and therefore reduce nutrient assimilation and biomass accumulation [9], [10]. In addition, petroleum pollution often results in altered microbial community structure and negatively influence consumption rate of soil resources, soil structure and water stress in petroleum-polluted soil [5], [12]–[14]. Petroleum pollution also intensifies competition between plants and microbes for nutrients during phytoremediation [5], [14].

Despite their important role in phytoremediation, surprisingly little is known about how plant–microbe interactions are influenced by petroleum pollution itself [5]. Better understanding of the impact of petroleum pollution on plant–microbe interactions is required to improve the sustainability and feasibility of phytoremediation [3]–[5], [15].

In this study, we used *Phragmites australis* as a phytoremedating species to examine how petroleum pollution affects plant ecophysiological traits, soil nutrient availability and microbe biology. This plant species is an excellent study organism owing to its potential of breaking down petroleum pollutants because of its well-developed roots, high biomass production, and stimulating effects for microbial degraders [6], [7], [14], [16], [17]. Due to the considerable impact of petroleum pollution on plants, soil properties, and microbes [11], [18]–[20], we hypothesized that petroleum pollution will negatively impact plant–microbe interactions.

## Results

### Effects of petroleum on plant traits

Linear regression analysis revealed significant effects of petroleum on 12 of 17 traits of *P. australis* measured at early stage of vegetative growth (Table S1 and Figure S1). Plant size (e.g., plant length, leaf length, and stem diameter), biomass (aboveground and belowground biomass), and potentially photosynthetic capacity (relative chlorophyll content) were negatively related to petroleum concentration (Table S1 and Figure S1). Furthermore, total carbon concentration (TC) of roots significantly decreased with increasing petroleum concentration, and total nitrogen concentration (TN) of leaves was positively correlated with petroleum concentration. At later stage of vegetative growth, petroleum also had significant impact on 10 of 17 plant traits of *P. australis* (Table S1 and Figure S1). In contrast to the earlier vegetative growth, TC of roots significantly increased with increasing petroleum concentration and TN of rhizomes and roots was positively correlated with petroleum concentration. Petroleum did not have significant effects on most of plant traits during the reproductive phase of the life cycle (Table S1 and Figure S1).

### Effects of petroleum on soil nutrients

**Figure 1:**
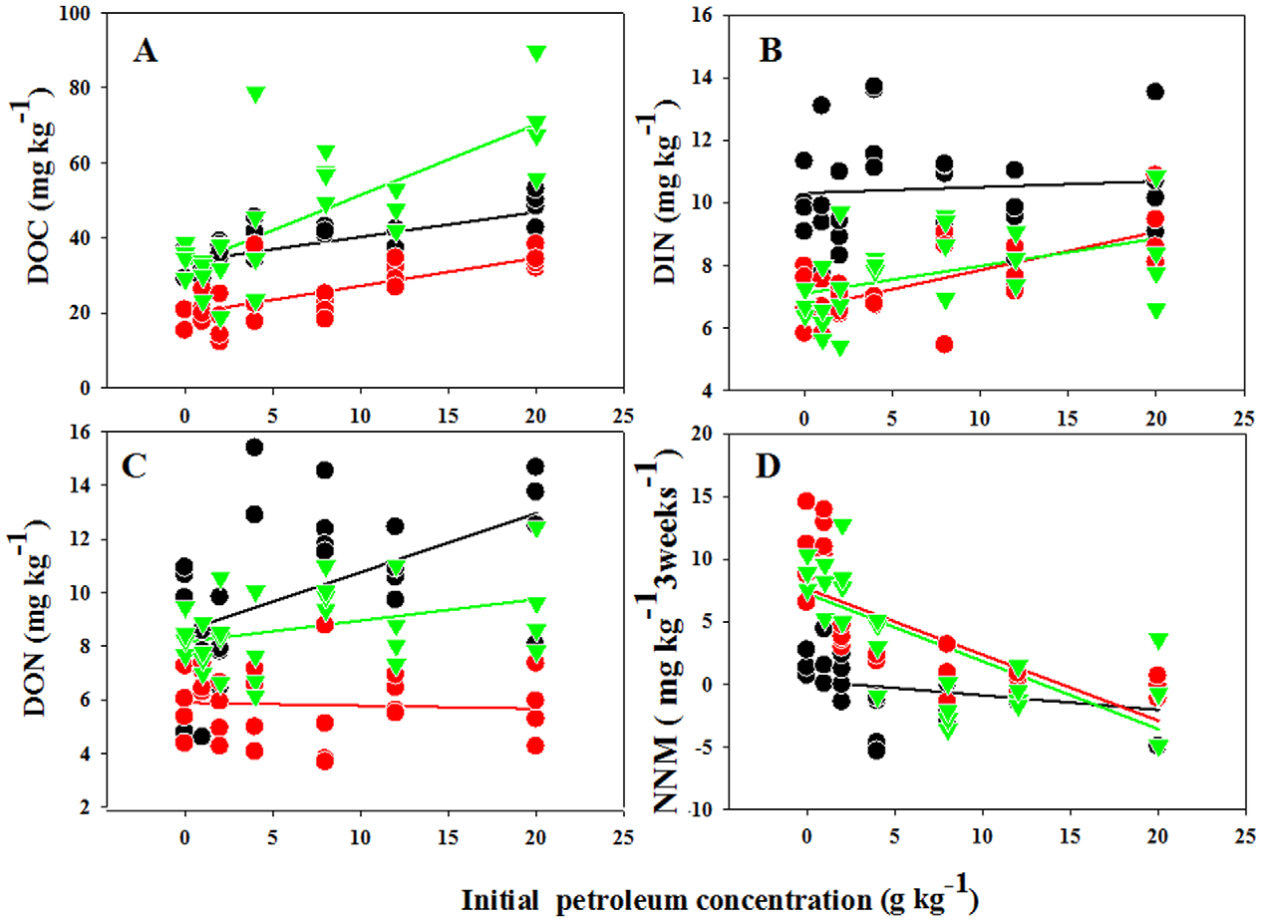
The effects of soil petroleum concentration on soil properties at early stage of vegetative growth (black circle), late stage of vegetative growth (red circle), and reproductive stage (green circle). The statistics of those regressions are listed in Table 1. DOC: dissolved organic carbon; DIN: dissolved inorganic nitrogen; DON: dissolved organic nitrogen; NNM: net nitrogen mineralization.

Our analyses showed that petroleum had significant positive effects on dissolved organic carbon concentration (DOC) across all plant growth stages, which tended to increase during the life cycle (Figure 1A and Table 1). DIN (dissolved inorganic nitrogen concentration) and DON had similar concentrations at different plant growth stages. DIN was significantly positively related with petroleum concentration at late vegetative growth and reproductive stages, while DON was positively correlated with petroleum concentration at the early stage of vegetative growth (Figure 1B, 1C, and Table 1). However, petroleum had significant negative effects on NNM at late stage of vegetative growth and reproductive stage (Figure 1D and Table 1). At the early stage of growth, the values of NNM were negative at high level of petroleum concentration, e.g., −1.834 mg kg^−1^ 3 weeks^−1^ at petroleum level of 8000 mg kg^−1^ (Figure 1D).

**Table 1:** Summary of regression analyses between petroleum concentration (X) and soil properties (Y).

Soil properties (Y)	Stage	*b*_1_	*b*_0_	*R^2^*	*P*	Soil properties (Y)	Stage	*b*_1_	*b*_0_	*R^2^*	*P*
DOC	EVG	0.659	33.703	0.506	<0.001	DON	EVG	0.219	8.575	0.26	<0.01
	LVG	0.746	19.795	0.444	<0.001		LVG	-0.011	5.896	0.004	NS
	REP	1.883	32.694	0.546	<0.001		REP	0.08	8.163	0.132	NS
	EVG *vs.* LVG	<0.001	NS				EVG *vs.* LVG	<0.001	<0.01		
	EVG *vs.* REP	<0.001	<0.01				EVG *vs.* REP	<0.001	NS		
	LVG *vs.* REP	<0.001	<0.01				LVG *vs.* REP	<0.001	NS		
DIN	EVG	0.019	10.307	0.006	NS	NNM	EVG	-0.117	0.277	0.127	NS
	LVG	0.122	6.625	0.461	<0.001		LVG	-0.527	7.612	0.523	<0.001
	REP	0.088	7.098	0.213	<0.05		REP	-0.542	7.232	0.536	<0.001
	EVG *vs.* LVG	<0.001	NS				EVG *vs.* LVG	<0.001	<0.001		
	EVG *vs.* REP	<0.001	NS				EVG *vs.* REP	<0.001	<0.001		
	LVG *vs.* REP	<0.001	NS				LVG *vs.* REP	<0.001	NS		

Equations are in the form Y  = *b*_1_X + *b*_0_. Differences among plant developmental stages were also tested using ANCOVA. NS means non-significant. EVG: the early stage of vegetative growth; LVG: the late stage of vegetative growth; and REP: the reproductive stage.

DOC: dissolved organic carbon; DIN: dissolved inorganic nitrogen; DON: dissolved organic nitrogen; NNM: net nitrogen mineralization.

### Effects of petroleum on soil enzymes and AMF colonization

**Figure 2:**
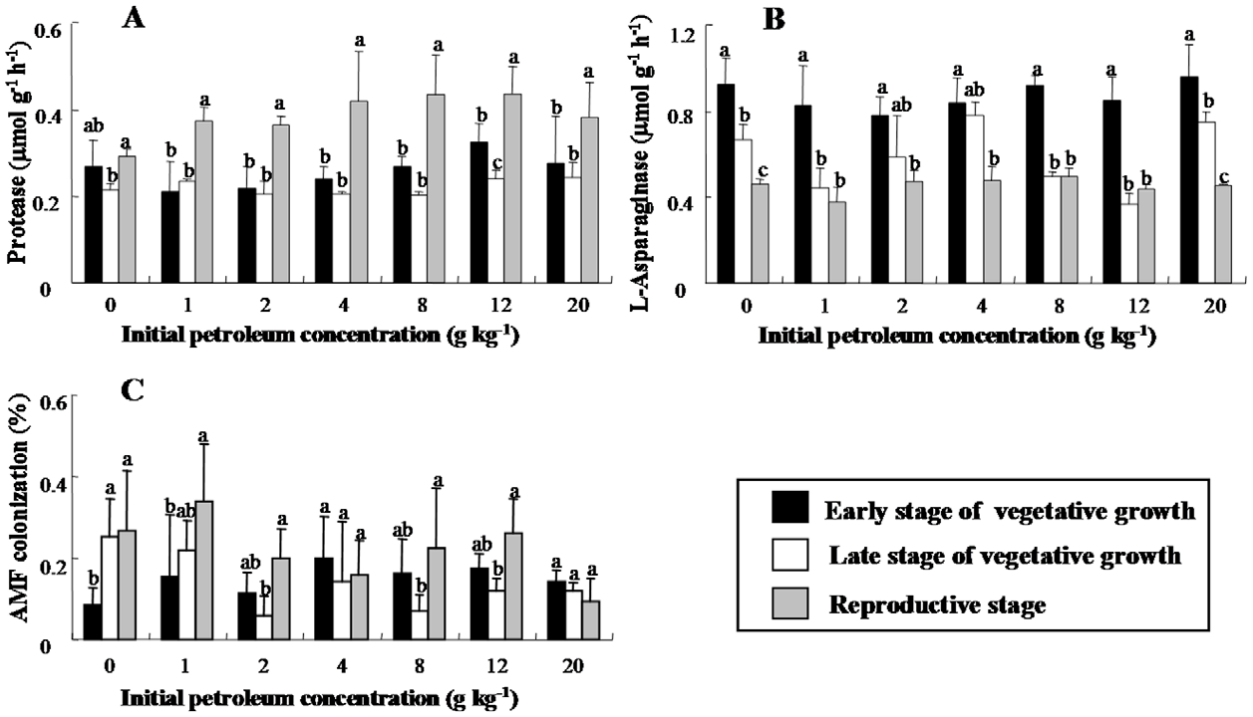
The effects of soil petroleum concentration on protease (A), L-asparaginase (B), and AMF colonization (C) at different plant growth stages. The vertical bars represent the standard deviations. The same lowercase letters denote non-significant difference between treatments (*P*>0.05).

Overall, plant growth stage had a greater influence on soil enzyme activities than petroleum concentration (Table 2). Protease activities showed significant changes corresponding to plant growth stages (Figure 2A and Table 2). Protease activities were generally higher at reproductive stage (0.270–0.585 µmol g^−1^ h^−1^) than at early (0.152–0.433 µmol g^−1^ h^−1^) and late (0.173–0.290 µmol g^−1^ h^−1^) stages of vegetative growth. L-Asparaginase activities also showed significant differences among the plant growth stages (Figure 2B and Table 2), being higher at early stage of vegetative growth (0.618–1.110 µmol g^−1^ h^−1^) than at late stage of vegetative growth (0.310–0.871 µmol g^−1^ h^−1^) and reproductive stage (0.329–0.566 µmol g^−1^ h^−1^).

**Table 2:** Summary of repeated measures ANOVA to test the effects of petroleum pollution, plant growth stage and their interaction (petroleum × stage) on protease, L-asparaginase and AMF colonization.

Parameters	Source of variation	Degrees of freedom (df1, df2)	F value	Significance
Protease	Petroleum	6,21	2.79	<0.05
	Stage	2, 20	61.43	<0.0001
	Petroleum × stage	12, 42	1.60	NS
L-Asparaginase	Petroleum	6, 21	5.34	<0.01
	Stage	2, 20	173.99	<0.0001
	Petroleum × stage	12, 42	3.07	<0.01
AMF colonization	Petroleum	6, 21	2.28	NS
	Stage	2, 20	6.67	<0.01
	Petroleum × stage	12, 42	2.20	<0.05

For the colonization of arbuscular mycorrhizal fungi (AMF), plant growth stage and its interaction with petroleum concentration significantly affected AMF colonization (Table 2), indicating that plant’s dependence on AMF varied among the plant growth stages. At early stage of vegetative growth, greater colonization was found in the roots from polluted soils (0.063–0.375%) than in those from unpolluted soils (0.034–0.125%) (Figure 2C). However, AMF colonization in the polluted soils at late stage of vegetative growth was generally lower than at early stage of vegetative growth, except for the slightly polluted soil (TPH = 1000 mg kg^−1^) (Figure 2C).

### Effects of petroleum on bacterial genes

**Figure 3:**
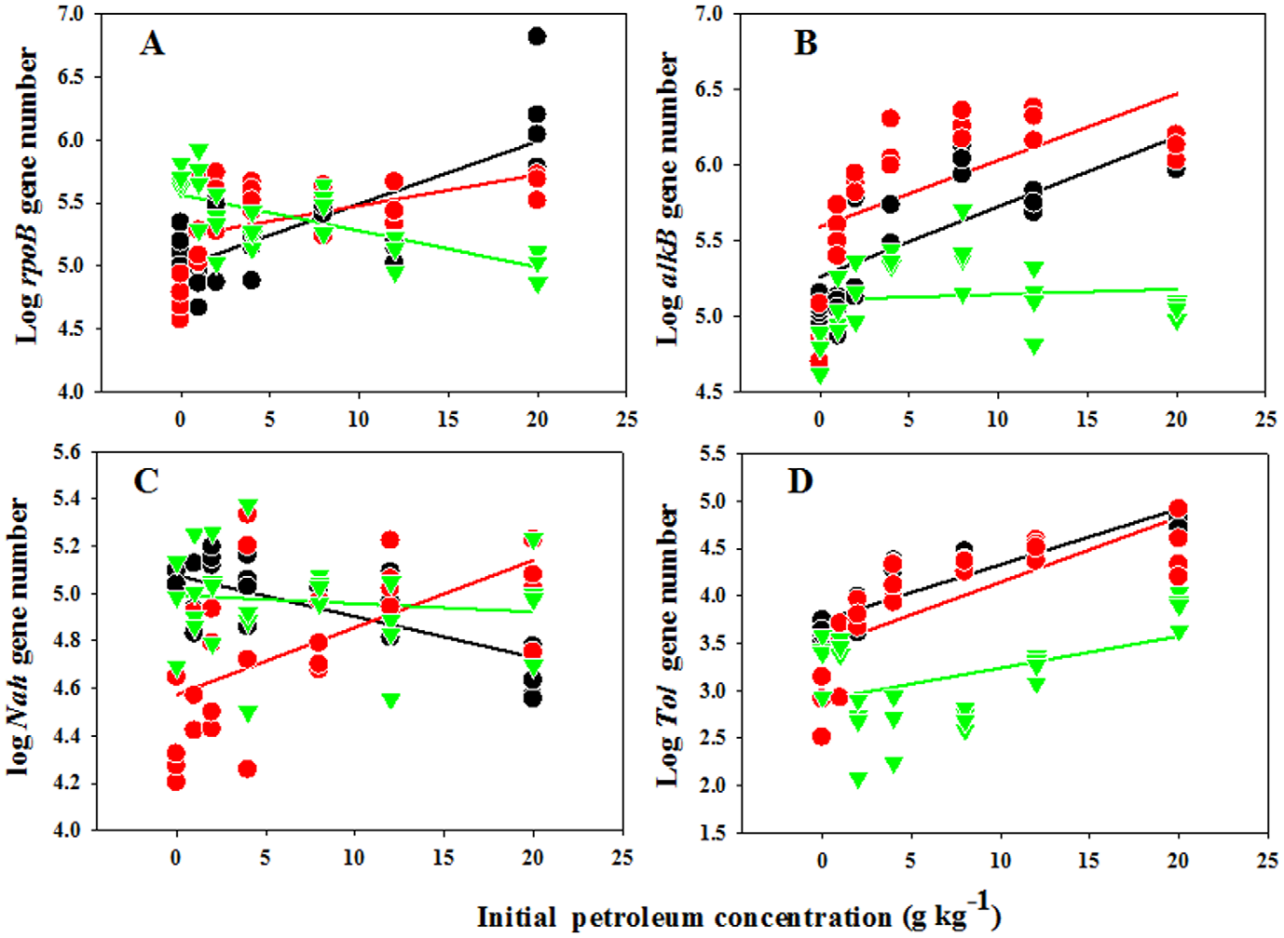
The effects of soil petroleum concentration on the numbers of microbial genes at early stage of vegetative growth (black circle), late stage of vegetative growth (red circle), and reproductive stage (green circle). The statistics of these regressions are listed in Table 2.

The number of four target genes examined had significant correlations with petroleum concentration across the plant growth stages. The number of *rpoB* gene increased with increasing petroleum concentration at both early and late vegetative growth stages, but the influence on *rpoB* gene was greater at early stage of vegetative growth than at late stage of vegetative growth (Figure 3A and Table 3). In contrast, petroleum had significant negative effects on *rpoB* gene at the reproductive stage.

**Table 3:** Summary of regression analyses between petroleum concentration (X) and copy number of microbial genes (Y). Equations are in the form Y =  *b*_1_X + *b*_0_.

Gene (Y)	Stage	*b*_1_	*b*_0_	*R^2^*	*P*	Gene (Y)	Stage	*b*_1_	*b*_0_	*R^2^*	*P*
*rpoB*	EVG	0.050	4.994	0.535	<0.001	*alkB*	EVG	0.046	5.262	0.547	<0.001
	LVG	0.025	5.231	0.247	<0.01		LVG	0.044	5.588	0.361	<0.001
	REP	-0.029	5.562	0.480	<0.001		REP	0.004	5.110	0.010	NS
	EVG *vs.* LVG	<0.05	<0.001				EVG *vs.* LVG	NS	<0.001		
	EVG *vs.* REP	<0.001	<0.001				EVG *vs.* REP	<0.001	<0.001		
	LVG *vs.* REP	<0.001	<0.001				LVG *vs.* REP	<0.01	<0.001		
*nah*	EVG	-0.017	5.076	0.475	<0.001	*tol*	EVG	0.059	3.740	0.794	<0.001
	LVG	0.028	4.573	0.351	<0.001		LVG	0.069	3.456	0.595	<0.001
	REP	-0.003	4.992	0.014	NS		REP	0.034	2.899	0.213	<0.05
	EVG *vs.* LVG	<0.001	<0.001				EVG *vs.* LVG	NS	<0.001		
	EVG *vs.* REP	<0.05	<0.001				EVG *vs.* REP	NS	<0.001		
	LVG *vs.* REP	<0.01	<0.001				LVG *vs.* REP	<0.05	<0.001		

Differences among plant developmental stages were tested using ANCOVA. NS means non-significant. EVG: the early stage of vegetative growth; LVG: the late stage of vegetative growth; and REP: the reproductive stage.

*rpoB*: the ribosomal polymerase B subunit gene; *alkB*: alkane monooxygenase gene; *nah*: naphthalene dioxygenase gene; *tol*: xylene monooxygenase gene.

Petroleum positively influenced the abundance of the *alkB* and *tol* genes across the plant growth stages (except for the *alkB* gene at reproductive stage) (Figure 3B, 3D and Table 3). For the *alkB* gene, a greater abundance was detected during later growth (Figure 3B and Table 3). In contrast, the *tol* gene was more abundant during the early stage of vegetative growth (Figure 3D and Table 3). The relationship between petroleum concentration and abundance was quite variable among life history stages for the *nah* gene: these were positive at early stage of vegetative growth, negative at late stage of vegetative growth (Figure 3C and Table 3) and there was no significant relationship at reproductive stage.

## Discussion

The major finding of this study is that petroleum pollution resulted in reduced plant performance. Plant size, biomass, and chlorophyll content (which represents potential photosynthetic capacity) were negatively related to petroleum concentration. Similar results were obtained from our previous study, showing that plant biomass significantly decreased under petroleum pollution during vegetative growth [21]. These relatively strong effects were mostly attributable to the major processes of plant growth and their interactions’ with soil environments that mainly occur during plant vegetative growth [15]. In addition, roots directly interact with soil biotic and abiotic factors and thus, root traits can vary more immediately in response to underground environments than other plant organs [22], [23]. TC of roots had different relationships with petroleum concentration at different plant growth stages (Table S1), suggesting that the responses of *P. australis* roots to petroleum pollution might be variable with plant development stages [24], [25].

Petroleum hydrocarbons often result in the degradation of plant-beneficial functions of microbes and high microbial consumption rate of nitrogen during phytoremediation [11], [19]. Therefore, nitrogen becomes the primary limiting nutrient for plant growth [11], [20]. Our results indicate that petroleum had consistently negative effects on NNM (Figure 1D and Table 1), which might reflect the decrease in the potential of microbes for supporting plant growth because NNM dominates inorganic nitrogen supply to the plants [26], [27].

Yet, we found that DIN (NH_4_^+^- N and NO_3_^−^-N) was not negatively influenced by petroleum and on the contrary, positive relationships between DIN and petroleum were observed during the later vegetative and reproductive stages (Figure 1B and Table 1). In addition, the activities of L-Asparaginase that plays an important role in nitrogen mineralization were more influenced by plant growth stage than by petroleum (Figure 2B and Table 2), suggesting that the supply of mineralized nitrogen by microbes to soils was more affected by plant growth stage than by petroleum pollution. Therefore, high consumption of mineralized nitrogen might have occurred during phytoremediation because petroleum had consistently negative effects on NNM. Our data suggest that greater abundances of total bacteria and hydrocarbon-degrading bacteria revealed by real-time PCR were generally found in petroleum-polluted soils in comparison to unpolluted soils (Figure 3 and Table 3). Thus microbial immobilization that removes inorganic nitrogen by microbial uptake may intensify nitrogen limitations to the plants due to higher consumption rate of nitrogen by a large number of hydrocarbon-degrading microbes during phytoremediation [11], [28]. The results obtained through ^15^N isotopic dilution technique also support the idea that the consumption rate of inorganic nitrogen is higher in petroleum-polluted soils than in unpolluted soils [29]. In unpolluted soils, microbial immobilization and mineralization rates are closely matched [29]. However, the immobilization rate was significantly higher than mineralization rate in petroleum-polluted soils [29].

DON contributed nearly 50% to the total extractable soil nitrogen (Figure 1B, 1C). This large amount of DON would likely constitute an important source of available nitrogen for plants when inorganic nitrogen could not meet the requirements for plant growth [30], [31]. Many studies suggest that DON is released from the soil through the action of microbial protease [32], [33]. In our study, plant growth stage had a greater influence on protease activities than petroleum pollution (Figure 2 and Table 2), and DON was positively correlated with protease activities at early stage of vegetative growth (*R^2^* = 0.39; *P*<0.05). These results imply that petroleum had limited effects on the supply of DON to plants at early stage of vegetative growth. On the other hand, through AMF-plant symbiosis, plants can compete for DON more efficiently with soil microbes because AMF acquires DON from the soil and exchanges DON with the host plants for photosynthetically derived carbohydrates that fuel fungal metabolism [30], [33]–[35]. In the present study, a higher rate of AMF colonization was found in plant roots from polluted soils compared to those from unpolluted soils at early stage of vegetative growth (Figure 2C). This appears to be an adaptive response of *P. australis* to petroleum pollution for uptake of DON because AMF colonization depends mainly upon their host plants to supply carbon [35], [36]. We used a labelling technique of stable isotope to assess how petroleum contamination affected *P. australis*’ use of different N forms, including DIN (^15^NH_4_^+^, ^15^NO_3_^−^) and DON (^13^C_2_-^15^N-Glycine) [29]. We found that the contribution of Glycine to the total of nitrogen assimilated by *P. australis* was greater in petroleum-polluted soils at early stage of vegetative growth [29]. Therefore, the potential of utilizing DON by *P. australis* at early stage of vegetative growth would play an important role in reducing initial nutrient deficiency in petroleum-polluted soil.

Although the efficiency of phytoremediation has been confirmed in many studies, little is known about how petroleum-degrading bacteria dynamically change with plant growth and development [3], [17]. In this study, we examined the number of three important catabolic genes that reflect the degradative potentials of bacterial communities [37]. Petroleum had significant positive effects on *alkB* and *tol* genes at both early and late stages of vegetative growth, indicated by the fact that the greater abundances of these two genes were also detected at both early and late stages of vegetative growth (Figure 3B, 3D, and Table 3). Our results suggest that petroleum mainly promotes the development of functional microbes containing *alkB* and *tol* genes during the vegetative growth of plants [18], [38]. These results show that the effectiveness of phytoremediation was plant-dependent, and that the interactions between plants and petroleum-degrading bacteria appeared to be relatively stronger during vegetative growth prior to the onset of reproduction [3], [4], [18]. On the other hand, a greater number of bacterial transcripts reflected by *rpoB* gene was detected in petroleum-polluted soil, suggesting petroleum also promoted the development of total bacterial community (Figure 3A and Table 3). Many studies have suggested that petroleum serves as growth substrates to petroleum-degrading bacteria, and hence enhances the abundances of petroleum-degrading bacteria [38]–[40]. Therefore, the increase in the number of hydrocarbon-degrading bacteria containing *alkB* and *tol* genes might be one of the reasons for enhancing total abundance of bacteria under petroleum pollution.

In contrast to the *alkB* and *tol* genes, the *nah* gene had different relationships with petroleum at plant vegetative growth stage (Figure 3C and Table 3). Previous studies have shown that the number of *nah* gene at high level of pollution is consistently greater than that at low level of pollution [37], [41]. Through enumeration of target genes by a real-time PCR-based assay, we found that different hydrocarbon-degrading bacteria had different dynamic changes during phytoremediation. For example, greater abundance of *alkB* was detected at the plant vegetative growth stages. Because phytoremediation of different components of petroleum is performed by different hydrocarbon-degrading bacteria, plants’ ability of phytoremediating different components of petroleum might be variable with plant growth and development. Phytoremediation might be most effective during the vegetative growth stages of plants as greater abundances of hydrocarbon-degrading bacteria containing *alkB* and *tol* genes were observed at these stages.

In summary, our study demonstrates that petroleum generally had negative impacts on *P. australis* traits. *P. australis* might enhance nitrogen availability by AMF colonization for coping with high consumption of mineralized nitrogen. This strategy is important to survival and establishment of plants in petroleum-polluted soils. The catabolic genes reflecting the degradative potential of bacterial communities showed that the plant vegetative growth stage is the more important phase for phytoremediation. Although our results obtained through our microcosm system might be different from those from very ‘old’ contaminated soils, lower nutrient availability might drive the plants to greater dependence upon the beneficial plant-microbe interactions for growth and development in nutrient-poor soils. Finally, the evidence obtained from this study indicates that utilizing plant-microbe interactions for phytoremediating petroleum-polluted soil requires a more comprehensive understanding of ecological linkages between aboveground and belowground processes. While our study contributes to the understanding of the roles of plant-microbe interactions in phytoremediating petroleum-polluted soils, field trials and in-depth mechanisms underlying phytoremediation still need to be further pursued.

## Materials and Methods

### Site and plant collection

All the experimental materials for this study were obtained in Shengli Oilfield in the Yellow River delta. The Shengli Oilfield is the second largest oil-producing base of China and lies in the Yellow River Delta (Shandong Province, eastern China, (37°33'N; 118°30'E)). Shengli Oilfield produces more than one hundred thousand tons of oily sludge per year and the maximum concentration of total petroleum hydrocarbon (TPH) in the soil is as high as 28.0 g kg^−1^ [7]. *P*. *australis* is one of the most dominant plant species in the oilfield, and covers more than 5% of the Yellow River Delta’s area [42]. This plant species has been chronically exposed to petroleum hydrocarbons since oil exploitation began in 1964 and might, to a certain degree, have adapted itself to the petroleum-polluted soils [43]. Our previous work has shown that *P*. *australis* has high potential for phytoremediation of petroleum-pollution at Shengli Oilfield, which removed TPH ( = 8.0 g kg^−1^) in soil by ∼75% compared to unplanted soils (30.3%) after one growing season [44]. We collected active rhizomes of *P*. *australis* from the center of a dense monoculture within a 3 m^2^ area so as to sample the rhizomes from the same clone.

### Soil sampling for microcosm experiment

The soil used in this study was collected from the topsoil (0–20 cm) at a site with no history of previous petroleum pollution at National Nature Reserve of the Yellow River Delta (37° 44 ' N; 118° 60 ' E) near the Shengli Oilfield [7]. After being transported to the laboratory, the soil was passed through a 1-mm sieve to remove plant residues and soil fauna, and then homogenized to give a composite sample (pH 7.23, TC 1.21%, TN 0.03%, DOC 64.74 mg kg^−1^, DIN 7.33 mg kg^−1^, DON 12.70 mg kg^−1^). Seven petroleum pollution treatments (0, 1.0, 2.0, 4.0, 8.0, 12.0, and 20.0 g kg^−1^) were designated by mixing the soil with crude oil supplied by the Shengli Oilfield.

### Microcosm experiment

The rhizomes that were collected in the field were cut into 5-cm-long segments and buried in partially-submerged garden soils in trays located in an unheated greenhouse. In late March, rhizome segments with clean roots and 10-cm-long shoots were randomly chosen as transplants for the microcosm experiment.

One rhizome segment was transplanted to each pot (16 cm in diameter and 20 cm in depth) which was filled with 2.5 kg of prepared soil (dry weight). A total of 84 pots (3 harvests (see below) ×7 treatments ×4 replicates) were randomly arranged in a polytunnel that received natural light in the Experimental Garden of Fudan University based in Shanghai, China (31° 18 ' N; 121° 30 ' E) in early April, 2008. Pots were watered every other day. In order to understand how the plant-microbe interactions might vary through the plant life cycle, we conducted three harvests corresponding to three stages during this experiment [45]: early vegetative growth (mid-June), late vegetative growth (mid-August), and sexual reproduction (mid-October).

The plots were randomly chosen and the plants were destructively harvested. After shoots were removed, each plot was divided into two equal subplots. Plant roots in one subplot were used to determine the colonization ratio of arbuscular mycorrhizal fungi (AMF), and the other to measure plant belowground traits. After removal of plant issues, the soil from a plot was mixed and then divided into two parts; one for determining soil nutrients and microbial enzyme activities (stored at 4°C), and the other for analyzing microbial genes by real-time PCR (Polymerase Chain Reaction) analysis (stored at −70°C). All samples were processed within 3 h after being collected.

### Plant traits

A total of 17 plant traits were measured in this study, including plant length, aboveground and belowground biomass, leaf length and width, relative chlorophyll content, stem diameter, internode length, tiller number, TC and TN of different plant tissues. Measuring methods for determining plant traits are listed in Table S2. These plant traits were chosen because of their physiological and morphological importance for plant growth and reproduction.

### Soil analysis

In order to determine the effects of petroleum pollution on soil nutrients, we measured soil DOC, DIN, DON, and NNM [46]. Soil DOC, DON and DIN were extracted by adding 25 g of each homogenized sample to 100 ml of 0.5 M potassium sulfate (K_2_SO_4_) and agitated on an orbital shaker table at 200 rpm for 1 h. NNM was estimated with 3-week aerobic incubation of 25 g of soil at 28°C, and calculated as the difference in the concentration of inorganic nitrogen in the incubated and initial samples. DIN in the K_2_SO_4_ extracts was calculated as the total concentration of NH_4_^+^-N and NO_3_^−^-N by using ammonia (No 1.14739.0001) and nitrate (No 1.09713.0001) test kit in a NOVA spectrophotometer (Merck, Germany). DOC and DON in the K_2_SO_4_ extracts were quantified by TOC/TN-analyzer (Shimadzu TOC-VCPH/TN, Kyoto, Japan). DON was calculated as TDN–DIN.

### Quantification of bacterial genes

A real-time PCR-based assay was used to quantify the magnitude of several target genes in sampled bacterial communities (*alkB* (alkane monooxygenase), *nah* (naphthalene dioxygenase) and *tol* (xylene monooxygenase). Samples were tested for catabolic and ribosomal genes using the PCR primers described previously [47], [48]. Briefly, one gram of soil from each microcosm was extracted by using the UltraClean Microbial DNA Kit (Mo Bio Laboratories, CA). Quantitative PCR was performed in 20 µl reaction mixtures using Platinum SYBR Green qPCR SuperMix (Invitrogen, CA) on an iQ5 thermocycler real-time PCR detection system (Bio-Rad). According to manufacturer recommended protocols with slight modifications, four target genes were numbered in the same cycling conditions as follows: holding for 3 min at 95°C, followed by 40 repeats of a 10-s denaturation step at 94°C, a 30-s annealing step at 55°C, and a 30-s extension step at 68°C. A melting curve analysis was performed after the final amplification period by using a temperature gradient from 68 to 94.5°C. Each DNA sample was tested in triplicate. The standard curves for the determination of gene numbers were established in the same manner described previously [47], [48].

### Soil enzymes and AMF colonization

To characterize soil microbial community functions, two soil enzyme activities relating the available nitrogen to plant performance (protease and L-asparaginase) were measured by adding different substrates. The protease activities were determined by measuring the products of tyrosine in the presence of the substrate sodium caseinate [49]; L-asparaginase activities were determined by measuring the products of ammonium in the presence of the substrate L-asparagines [32]. Analyses were conducted in triplicate for each sample. A control was used with a soil and buffer mixture in the absence of the substrate to account for the background level. Enzyme activity is expressed as µmol g^−1^ h^−1^.

AMF colonization that may affect nutrient acquisition of host plants was determined as described previously [50]. The fine roots (<1 mm) were selected and stained by glycerol-trypan blue solution. The stained root samples were examined at 45–100× magnification and quantification of root colonization by AMF was estimated by the gridline-intersect method [51].

### Data analysis

Linear regression was used to examine the effects of petroleum pollution on plant traits, soil nutrients and bacterial genes at different stages of plant growth. The data on bacterial genes were log(x) transformed prior to regression analysis where necessary to linearize the relationships. To control for variations in petroleum concentrations at different harvesting times, the effects of plant growth stage on soil nutrients and the numbers of target genes were tested by analysis of covariance (ANCOVA). ANCOVAs were applied to test the differences in the slope (*b*_1_) and intercept (*b*_0_) of linear regressions (Y =  *b*_1_X + *b*_0_) among the plant growth stages. Additionally, repeated-measures ANOVA was used to test for the effects of petroleum pollution, plant growth stage and their interaction on protease and L-asparaginase activities, and AMF colonization. Tukey’s test was used to determine a posteriori differences at *P* < 0.05. All of the statistical analysis was performed with the software SPSS 13.0.

## Supporting Information

Figure S1 **The effects of soil petroleum concentration on plant traits at different plant growth stages.**
(DOCX)

Table S1 **Summary of correlations of oil concentration with plant traits.**
(DOCX)

Table S2 **Measuring methods for determining plant traits.**
(DOCX)
