# Supplementary material for: Understanding Plant-Microbe Interactions for Phytoremediation of Petroleum-Polluted Soil
Source: PLoS One. 2011 Mar 18;6(3):e17961. doi: 10.1371/journal.pone.0017961 (PMC3060916; doi:10.1371/journal.pone.0017961)
Supplement: Figure S1 — The effects of soil petroleum concentration on plant traits at different plant growth stages. (DOCX) [file pone.0017961.s001.docx]

**Figure S1** The effects of soil petroleum concentration on plant traits at different plant growth stages.
