# Supplementary material for: Understanding Plant-Microbe Interactions for Phytoremediation of Petroleum-Polluted Soil
Source: PLoS One. 2011 Mar 18;6(3):e17961. doi: 10.1371/journal.pone.0017961 (PMC3060916; doi:10.1371/journal.pone.0017961)
Supplement: Table S1 — Summary of correlations of oil concentration with plant traits. (DOCX) [file pone.0017961.s002.docx]

**Table S1** Summary of correlations of plant traits (Y) to oil concentration (X). The regression equations are in the form Y = *b*_1_X + *b*_0._ NS means non-significant.

| Plant traits (Y) | Early stage of vegetative growth | | | | Late stage of vegetative growth | | | | Reproductive stage | | | |
| --- | --- | --- | --- | --- | --- | --- | --- | --- | --- | --- | --- | --- |
|  | *b*_1_ | *b*_0_ | *R^2^* | *P* | *b*_1_ | *b*_0_ | *R^2^* | *P* | *b*_1_ | *b*_0_ | *R^2^* | *P* |
| Plant length^a^ | -1.87 | 92.54 | 0.77 | <0.001 | -0.85 | 45.93 | 0.17 | <0.05 | -0.53 | 22.91 | 0.09 | NS |
| Aboveground biomass^a^ | -0.19 | 4.67 | 0.81 | <0.001 | -0.56 | 12.90 | 0.57 | <0.001 | -0.08 | 3.55 | 0.02 | NS |
| Belowground biomass^a^ | -0.06 | 1.65 | 0.37 | <0.001 | -0.24 | 6.84 | 0.49 | <0.001 | -0.14 | 4.86 | 0.07 | NS |
| Leaf length^a^ | -0.39 | 24.50 | 0.57 | <0.001 | 0.14 | -0.67 | 0.08 | NS | -0.03 | -8.11 | 0.00 | NS |
| Leaf width^a^ | -0.03 | 1.59 | 0.72 | <0.001 | -0.12 | 2.21 | 0.31 | <0.01 | -0.24 | -1.92 | 0.35 | <0.001 |
| Stem diameter^a^ | -0.08 | 3.16 | 0.70 | <0.001 | -0.04 | 1.76 | 0.15 | <0.05 | 0.08 | -1.45 | 0.34 | <0.01 |
| Internode length^a^ | -0.27 | 9.56 | 0.67 | <0.001 | 0.21 | -1.15 | 0.49 | <0.001 | -0.05 | -1.80 | 0.07 | NS |
| Tiller number^a^ | -0.22 | 6.84 | 0.50 | <0.001 | -0.08 | 3.79 | 0.05 | NS | 0.13 | 1.30 | 0.03 | NS |
| Relative chlorophyll content | -0.24 | 41.39 | 0.41 | <0.001 | -0.42 | 33.93 | 0.35 | <0.001 | -0.38 | 36.18 | 0.37 | <0.001 |
| Total carbon of leaf | -0.13 | 44.12 | 0.16 | <0.05 | -0.04 | 44.82 | 0.04 | NS | 0.13 | 43.98 | 0.34 | <0.01 |
| Total carbon of stem | -0.05 | 41.02 | 0.02 | NS | -0.03 | 44.16 | 0.03 | NS | -0.03 | 44.98 | 0.07 | NS |
| Total carbon of rhizome | -0.03 | 40.38 | 0.01 | NS | 0.07 | 42.02 | 0.12 | NS | -0.08 | 45.06 | 0.42 | <0.01 |
| Total carbon of root | -0.23 | 37.07 | 0.36 | <0.001 | 0.20 | 37.08 | 0.57 | <0.001 | 0.06 | 41.70 | 0.11 | NS |
| Total nitrogen of leaf | 0.02 | 2.67 | 0.18 | <0.05 | 0.00 | 1.71 | 0.02 | NS | 0.01 | 2.00 | 0.06 | NS |
| Total nitrogen of stem | 0.00 | 1.38 | 0.01 | NS | 0.00 | 0.48 | 0.00 | NS | 0.01 | 0.32 | 0.28 | NS |
| Total nitrogen of rhizome | 0.01 | 0.91 | 0.01 | NS | 0.01 | 0.38 | 0.15 | <0.05 | 0.00 | 0.42 | 0.03 | NS |
| Total nitrogen of root | -0.01 | 1.34 | 0.09 | NS | 0.01 | 0.69 | 0.42 | <0.001 | 0.01 | 0.86 | 0.10 | NS |

^a^ The independent variable (Y) in the regression analysis were their increment at each plant developmental stage relative to the previous one.
