# Supplementary material for: Understanding Plant-Microbe Interactions for Phytoremediation of Petroleum-Polluted Soil
Source: PLoS One. 2011 Mar 18;6(3):e17961. doi: 10.1371/journal.pone.0017961 (PMC3060916; doi:10.1371/journal.pone.0017961)
Supplement: Table S2 — Measuring methods for determining plant traits. (DOCX) [file pone.0017961.s003.docx]

**Table S2** Measuring methods for determining plant traits

| Plant trait | Explanation | Units |
| --- | --- | --- |
| Plant length | Length from the ground to the tip of the plant (bent plants pulled up; mean of the first three highest individuals ) | cm |
| Aboveground/belowground biomass | Dry weight of plant aboveground / belowground biomass (oven-dried at 70 °C for 72 h ) | g |
| Leaf length/width | Length and width of leaf blade (mean of three topmost leaves of the first three highest individuals) | cm |
| Relative chlorophyll content | Measure by using a chlorophyll meter, Minolta SPAD-502, which gives a value that is well correlated with chlorophyll content (mean of three topmost leaves of the first three highest individuals) | % |
| Stem diameter/internode length | Diameter and length of internodes (from the 2th to the 3th node) along the stem from the ground ( mean of the first three highest individuals) | mm, cm |
| Tiller number | The number of shoots per microcosm after tillering | individual |
| Total carbon/nitrogen concentrations of different tissues | Total carbon / nitrogen concentrations of plant leaf, stem, root, and rhizome (ground to powder, and then analyzed by a N/C Analyzer, Flash EA 1112 Series; Thermo Finnigan) | % |
